# Endoscopic-assisted linea alba reconstruction: New technique for treatment of symptomatic umbilical, trocar, and/or epigastric hernias with concomitant rectus abdominis diastasis

**DOI:** 10.1007/s10353-017-0473-1

**Published:** 2017-03-10

**Authors:** Ferdinand Köckerling, Marinos Damianos Botsinis, Christine Rohde, Wolfgang Reinpold, Christine Schug-Pass

**Affiliations:** 1 Department of Surgery and Center for Minimally Invasive Surgery, Academic Teaching Hospital of Charité Medical School, Vivantes Hospital, Neue Bergstrasse 6, 13585 Berlin, Germany; 2 Department of Surgery and Hernia Center, Wilhelmsburg Hospital Gross-Sand, Gross-Sand 3, 21107 Hamburg, Germany

**Keywords:** Rectus abdominis diastasis, Umbilical hernia, Epigastric hernia, Trocar hernia, Mesh augmentation, Complications

## Abstract

**Background:**

Patients with symptomatic umbilical, trocar, and/or epigastric hernias and concomitant rectus abdominis diastasis represent a growing clinical problem. The optimal management of this complex hernia situation is the subject of debate in the literature. This paper reports the early results of an innovative surgical technique aimed at managing this hernia situation.

**Methods:**

Endoscopic-assisted linea alba reconstruction (ELAR) with mesh augmentation is a surgical technique long known in the literature for its good outcome for incisional hernia repair (myofascial release, overlapping herniorrhaphy, Gibson’s operation, shoelace repair, anterior rectus sheath repair, dynamic patch plasty) via a small access route. The early results for 140 patients are presented here.

**Results:**

Two patients (1.4%) developed postoperative complications requiring redo surgery. These were two cases of diffuse secondary bleeding without an identifiable bleeding source, in one patient with liver cirrhosis and portal hypertension and in another patient receiving treatment with platelet aggregation inhibitors. All other complications were successively managed with conservative treatment. After 1 year, two of 30 patients reported occasional pain, including pain at rest in one patient.

**Conclusion:**

The ELAR technique with mesh augmentation is an innovative, minimally invasive surgical procedure for treatment of patients with a complex abdominal wall hernia comprising symptomatic umbilical, trocar, and/or epigastric hernias with concomitant rectus abdominis diastasis.

## Introduction

Rectus abdominis diastasis (RAD) describes a condition in which the two rectus muscles are separated by an abnormally wide distance of more than 2 cm [1]. When a patient with RAD raises his or her head and begins to sit up, the increase in intra-abdominal pressure as the two rectus muscles contract can result in a diffuse fusiform bulge [1]. Patients with RAD typically are middle-aged and older men with central obesity, or small, fit women who have carried a large fetus or twins to term [1]. Rectus abdominis diastasis is often seen in umbilical and/or epigastric hernia (45%) [2]. Patients with small umbilical and/or epigastric hernia with concomitant RAD, who underwent suture repair, had a significantly higher recurrence rate (31.2% vs. 8.3%; *p* < 0.001) [2]. The authors concluded that umbilical and/or epigastric hernias, regardless of size, with concomitant RAD require mesh repair owing to unacceptably higher recurrence rates [2]. In full-extend RAD instead of a stable linea alba only a very thin membrane extends longitudinally from the xiphoid process to the subumbilical area, also extending laterally on both sides by several centimeters lateral to the midline. Accordingly, anatomic reconstruction with mesh augmentation is needed for effective repair and to prevent recurrence of not only the umbilical or epigastric hernia but also of RAD.

If RAD is symptomatic or associated with midline hernias (umbilical, trocar and/or epigastric), corrective surgery of all pathologies at the same time could represent the most recommended approach [3].

Owing to missing data, it was not possible in a systematic review to find any clear evidence of the superiority of an endoscopic or open technique for the treatment of RAD with concomitant umbilical and/or epigastric hernia [4].

The spectrum of surgical procedures ranges from the open sublay technique through open myofascial release with and without mesh to laparo-endoscopic techniques [5]. Furthermore, there are innovative techniques such as the EMILOS and MILOS operations [6, 7].

Another alternative is the endoscopic-assisted linea alba reconstruction (ELAR), which is a hybrid technique [5]. However, this surgical technique is not new but is based on the long-established method reported on with good results in the literature under various names such as “myofascial release” [8], “overlapping herniorrhaphy”, “onlay prosthesis” [9, 10], “shoelace repair” [11], “Gibson’s operation” [12, 13], “modified shoelace repair” [14], “anterior rectus sheath repair” [15], ”dynamic patch plasty” [16], and “autodermal hernioplasty”, as described by Rehn [17]. A common feature of all these techniques is that the anterior layer of the rectus sheath is exposed from the xiphoid process to the subumbilical area and then incised. Next, the medial segments of the anterior layer of both rectus sheaths are sutured together at the midline for reconstruction of a new linea alba. This also closes the defects caused by ventral and incisional hernias. Then the resultant defect in the anterior layer of the rectus sheath is repaired by suturing a mesh for augmentation of the anatomic reconstruction.

The authors have reported good to very good results on using this technique for incisional hernia. It should therefore also be suitable for anatomic reconstruction of the abdominal wall in association with umbilical, trocar, and/or epigastric hernias and concomitant RAD. With the addition of video-endoscopic equipment, this operative procedure was further developed to a hybrid technique to optimize the results obtained with as small as possible an access route [5]. The operative technique and early results of the ELAR operation are presented here.

## Methods

### Operative technique

**Fig. 1:**
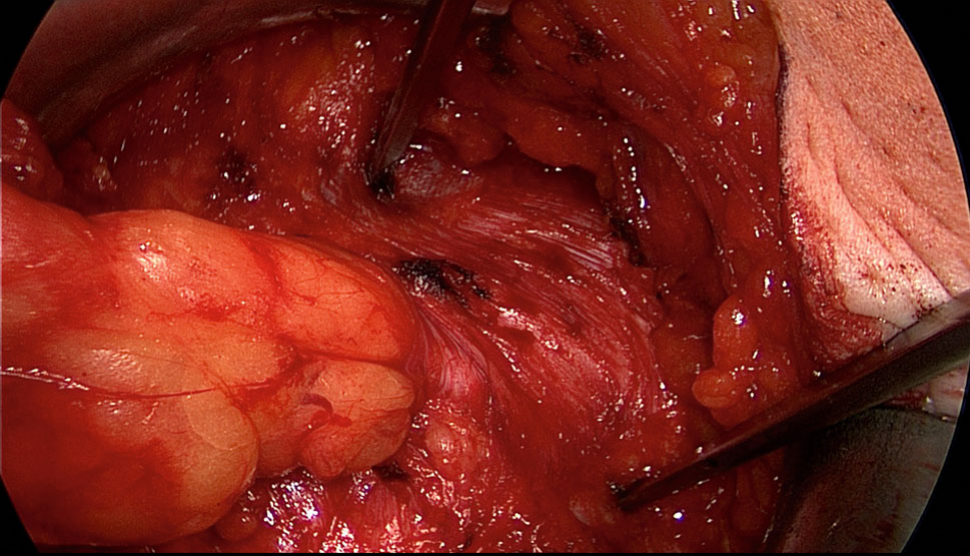
Characteristic findings of epigastric hernia and concomitant RAD. The opened forceps shows rectus abdominis diastasis with width of 6 cm

**Fig. 2:**
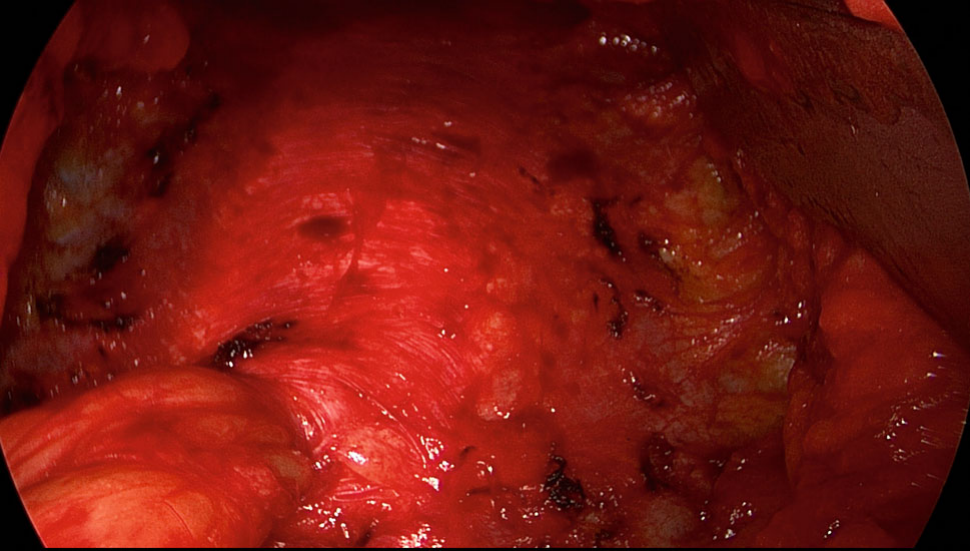
Following a combination of blunt and sharp dissection of the subcutaneous tissue, the fine RAD membrane can be identified as a remnant of the former linea alba

**Fig. 3:**
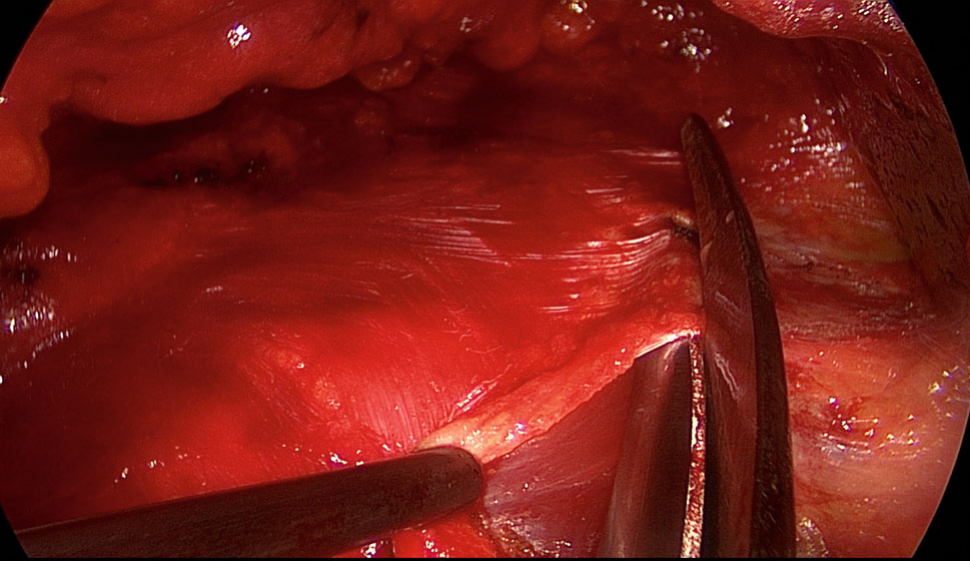
Incision made with scissors in the anterior layer of rectus sheath, on the left

**Fig. 4:**
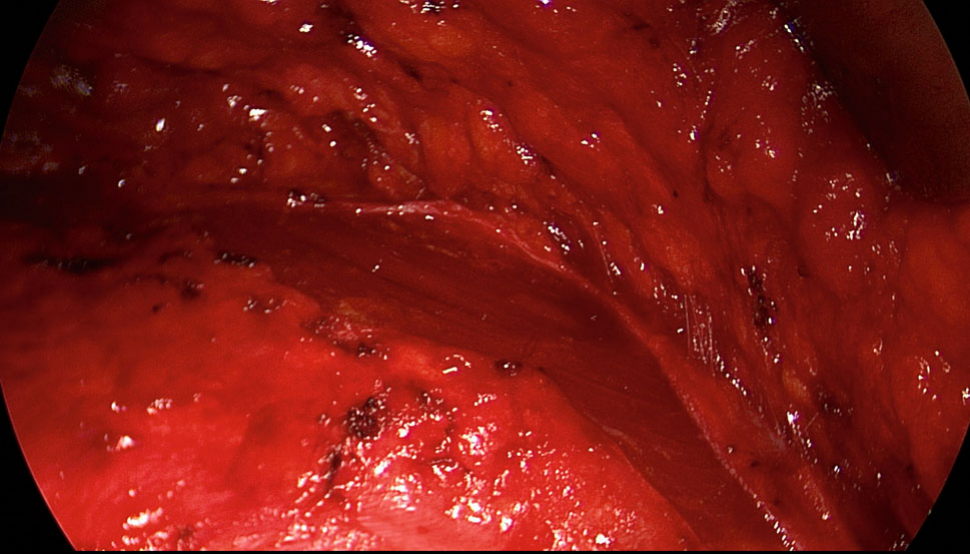
Bilateral exposure of the rectus muscle extending from the xiphoid process to several centimeters below the umbilicus

**Fig. 5:**
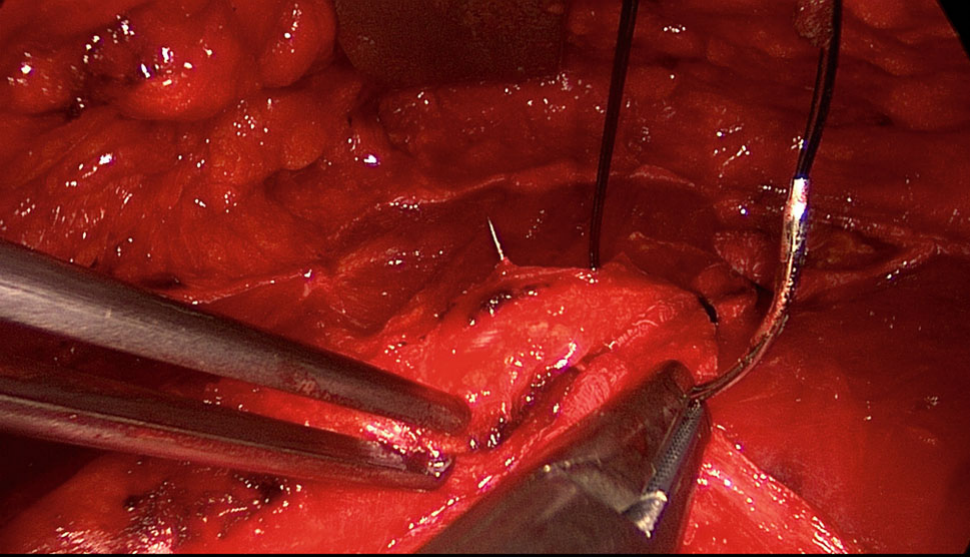
Following incision of the anterior rectus sheath on both sides, the two medial segments of the anterior layer of the rectus sheath are sutured together at the midline using non-absorbable loop suture

**Fig. 6:**
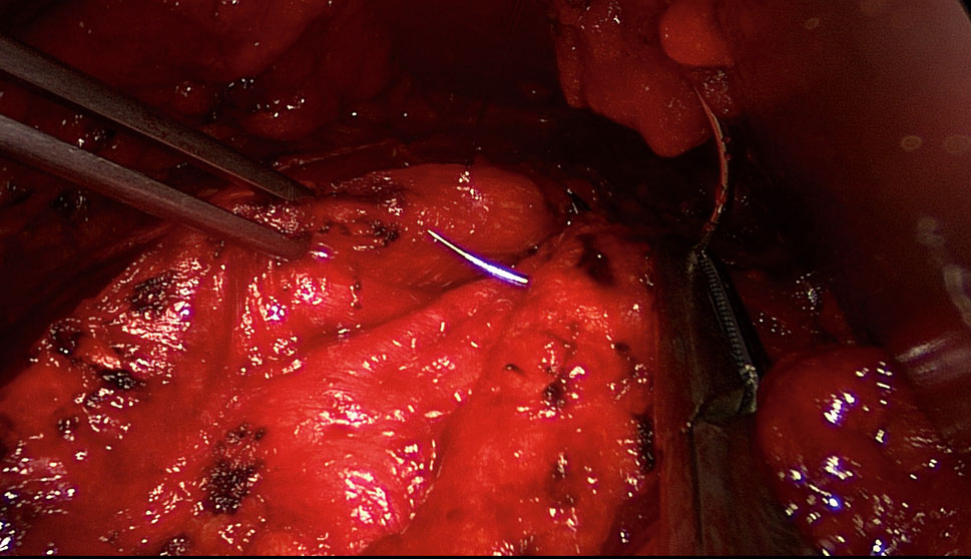
Inward plication of RAD with suture

**Fig. 7:**
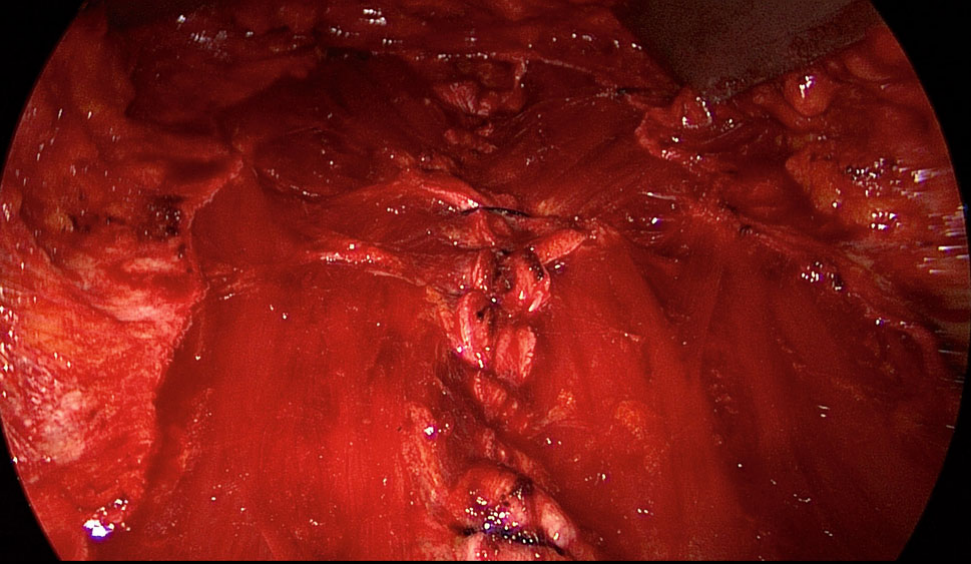
A new linea alba is formed once suturing is complete, all defects and RAD are closed and the rectus muscles are restored to their position at the midline adjacent to the reconstructed linea alba

**Fig. 8:**
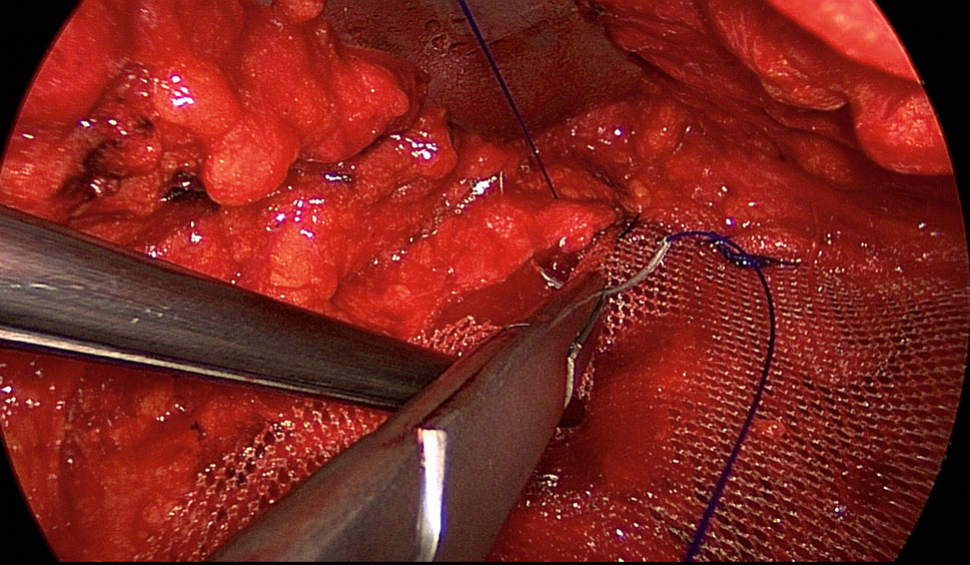
Fixation of polypropylene mesh (TiMesh strong) with continuous, non-absorbable sutures at the incision margin of the anterior layer of the rectus sheath

**Fig. 9:**
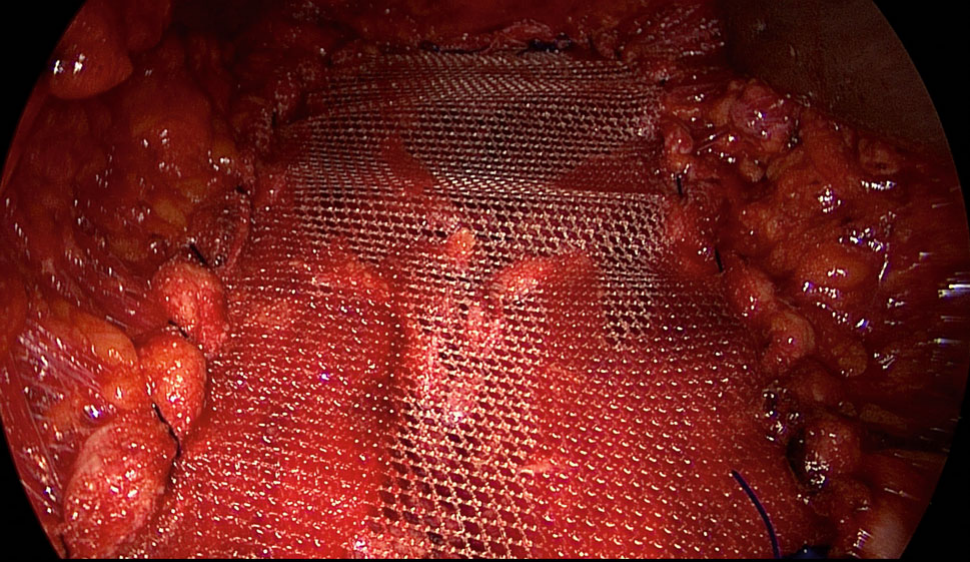
Stable augmentation of defect closure through myofascial release and linea alba reconstruction

**Fig. 10:**
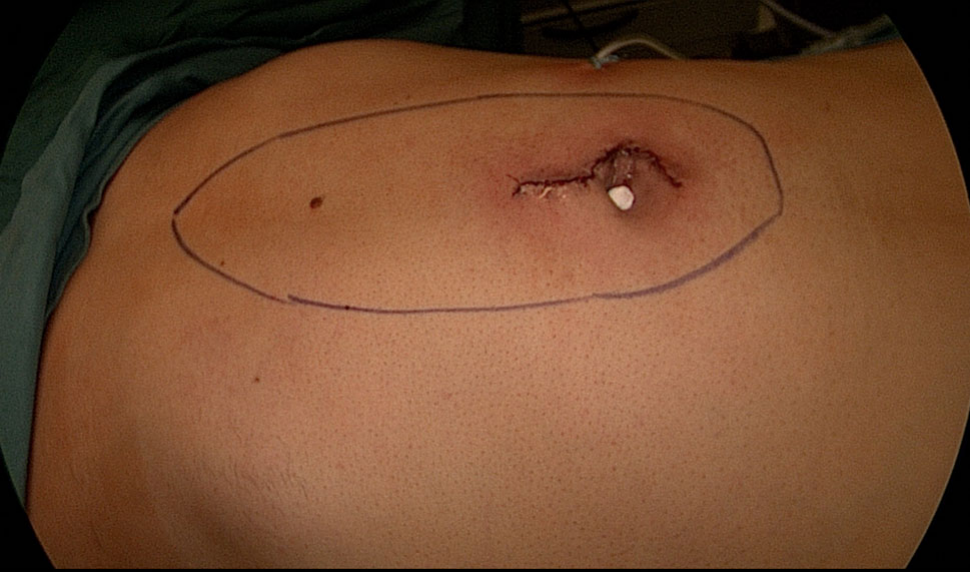
Status post placement of a subcutaneous Redon drain and intracutaneous skin suture

The patient is positioned supine with the left arm tucked at the side and the right arm abducted. The video-endoscopic equipment is positioned to the left of the patient. The video-endoscopic equipment needed includes the camera, optics, and light source. The patient is given preoperative single-shot antibiotic prophylaxis. The entire abdomen is thoroughly sterilized and draped (Fig. 10). The access route consists of a half loop on the left around the umbilicus, extending 2–3 cm cranially in the midline (Fig. 10). Diathermy dissection of the subcutaneous tissue is performed, already revealing in most cases the umbilical, trocar, and/or epigastric hernia (Fig. 1). Any fatty tissue in the hernia sac can be removed with a clamp. Circular exposure of an umbilical hernia is performed, and the hernia sac is opened and resected at the hernia margin, thus separating the umbilicus from the fascia. The hernia sac content of an umbilical hernia are either repositioned or likewise resected. Next, depending on the RAD clinical findings, the anterior layer of the rectus sheath is exposed on both sides from the xiphoid process and extends several centimeters below the umbilicus. Now the wafer-thin RAD membrane, the former linea alba, can be identified (Fig. 2). The anterior layer of the rectus sheath is detached on both sides with a width of around 4–5 cm from the subcutaneous tissue. Using the optics and the video-endoscopic equipment light source, dissection is performed superior to the skin incision beneath the abdominal skin and the subcutaneous tissue and the anterior rectus sheath. The surgeon has a direct view of the surgical area via the skin incision, but needs the light source to that effect, while the two assistants watch the monitor of the video-endoscopic equipment positioned to the right of the patient. Once the anterior layers of both rectus sheaths have been adequately exposed, an incision is made with a scissors around 2 cm from the medial margin of the rectus sheath (Fig. 3). Again, the incision runs bilaterally from the xiphoid process to the subumbilical area, thus exposing the bellies of both rectus muscles (Fig. 4). After that, the abdominal wall is restored to its normal anatomy. To that effect, the two resected medial segments of the anterior layer of the rectus sheath are sutured together using continuous, non-absorbable loop sutures (Fig. 5). This can be accomplished with relatively little tension thanks to myofascial release. Inward plication of the RAD is effected (Fig. 6). A new linea alba is formed once suturing is complete, all defects and the RAD are closed, and both rectus muscles are restored to their position at the midline adjacent to the reconstructed linea alba (Fig. 7). This is followed by placement of a polypropylene mesh for augmentation of myofascial release. The mesh is first optimally tailored to the size of the defect in the anterior layers of the rectus sheaths. Only then is the mesh sutured to the incision margin of the anterior layer of the rectus sheath using continuous non-absorbable suturing material. The depth of the fascial sutures should be at least 1 cm throughout to assure secure mesh fixation (Fig. 8). Following bilateral, circular mesh fixation to the incision margin of the anterior layer of the rectus sheath, stabile augmentation of the defect and RAD closure following myofascial release and reconstruction of the linea alba are assured (Fig. 9). Next, a Redon drain is inserted between the mesh and subcutaneous tissue (Fig. 10). Once the subcutaneous tissue is sutured, the skin is closed with an intracutaneous suture (Fig. 10). Patients wear an abdominal binder for 6 weeks after the operation. The drain is removed once 24-h fluid production is less than 30 ml, as is generally the case after 3 days.

### Patients

Between 15 June 2015 and 1 January 2017, a total of 140 patients were operated on with this technique. These were 90 men and 50 women. All patients signed an informed consent. As the described operation technique was performed for many years via larger incision in our hospital and results are reported in many publications in the literature, approval by an ethics committee was not necessary.

The mean age was 54.7 years with range of 25–85 years. The indications were RAD with symptomatic umbilical hernias (*n* = 17), epigastric hernias (*n* = 8), umbilical and epigastric hernias (*n* = 75), and incisional hernias (*n* = 40), which were mainly trocar hernias resulting from periumbilical insertion of a 10-mm trocar during laparoscopic operation. The patients had a mean body mass index (BMI) of 29.9 with range of 18–50. The mean defect length was 8.6 cm (range: 3–15 cm) and the defect width, 5.9 cm (range: 4–11 cm).

## Results

The mean operating time was 116 min (range: 62–209 min). The meshes used had a mean length of 18.7 cm (range: 13–28 cm) and mean width of 9.1 cm (range: 5–15 cm). The mean hospital stay was 4.5 days (range: 2–18 days). Two patients experienced postoperative secondary bleeding requiring redo surgery (1.4%). In each case this involved diffuse secondary bleeding without an identifiable bleeding source, in one patient with liver cirrhosis and portal hypertension and in another patient receiving treatment with platelet aggregation inhibitors. Nine patients (6.4%) developed discrete impaired wound healing or discrete necrosis adjacent to the incision; they all responded to conservative treatment. In all, 125 of the 140 patients who had undergone surgery already attended the scheduled follow-up review 30 days after the operation. At that time two patients (1.6%) still showed evidence of discrete impaired wound healing, which was further treated by us. Six patients (4.8%) had a seroma of no clinical significance and which required no treatment. Twenty-six patients (20.8%) still reported intermittent pain in the region of the mesh margins. Only seven patients continued to take painkillers (5.6%).

Thirty patients have already attended their 1‑year follow-up examination. There was no evidence of seroma, impaired wound healing, or recurrence. Two patients still reported occasional pain, including pain at rest in one patient.

## Discussion

Symptomatic RAD is a relatively common change in the abdominal wall, which has no pathological significance. However, any concomitant development of umbilical hernias, epigastric hernias, and trocar hernias often leads to symptomatic RAD. This then raises the question of optimal reconstruction for management of this complex hernia situation. Open operations via large access routes find little acceptance among patients. With this constellation of findings, endoscopic techniques have their limitations since endoscopic repair of RAD-mediated bulging is difficult. Alternative techniques include the MILOS and EMILOS innovations [6, 7]. And as a further innovative technique, the ELAR operation with its early results are described here [5]. It embodies a technique long described under various names in the literature for incisional hernia repair via a relatively small access route. The advantage bestowed by this technique is that it restores the abdominal wall anatomy through the use of autologous tissue and additional mesh augmentation for stabilization of the reconstruction. This technique eliminates RAD, which was the underlying cause of the abdominal wall defects. It also restores the functions of the rectus muscles that are able to resume such functions as stabilizers of the trunk only when they are positioned adjacent to the new linea alba. The early postoperative results up to 30 days revealed a complication-related redo surgery rate of only 1.4%. All other postoperative complications responded to conservative treatment. Likewise, the initial findings after 1 year, albeit only available so far for a relatively small number of patients, demonstrated encouragingly low recurrence and pain rates. Naturally, these findings must be confirmed on the basis of a larger number of cases and further studies.

## Conclusion

Endoscopic-assisted linea alba reconstruction (ELAR) is a promising operative technique, in particular for the patient group with RAD and concomitant umbilical, trocar, and epigastric hernias since it can be implemented via a relatively small open access route, thus reducing the complication rate. This is confirmed by the results presented here. However, further studies with a greater number of cases and longer follow-up are needed before final assessment of this surgical technique can be made.
